# N-acetylcysteine-functionalized coating avoids bacterial adhesion and biofilm formation

**DOI:** 10.1038/s41598-017-17310-4

**Published:** 2017-12-12

**Authors:** Fabíola Costa, Daniela M. Sousa, Paula Parreira, Meriem Lamghari, Paula Gomes, M. Cristina L. Martins

**Affiliations:** 1 i3S, Instituto de Investigação e Inovação em Saúde, Universidade do Porto, Rua, Portugal; 2 INEB - Instituto de Engenharia Biomédica, Universidade do Porto, Porto, Portugal; 3 UCIBIO-REQUIMTE, Departamento de Química e Bioquímica, Faculdade de Ciências, Universidade do Porto, Porto, Portugal; 4 Universidade do Porto, Instituto de Ciências Biomédicas Abel Salazar, Porto, Portugal

## Abstract

N-acetyl cysteine (NAC) is an FDA-approved drug clinically applied on a broad range of pathologies. Further research has been conducted with this drug to benefit from its antimicrobial activity potential. However, NAC has a very short half-life and therefore strategies that accomplish high local concentrations would be beneficial. In this study, covalent immobilization of NAC was performed, in order to obtain long-lasting high local concentration of the drug onto a chitosan(Ch)-derived implant-related coating. For the development of NAC-functionalized Ch films, water-based carbodiimide chemistry was applied to avoid the use of toxic organic solvents. Here we report the optimization steps performed to immobilize NAC onto the surface of pre-prepared Ch coatings, to ensure full exposure of NAC. Surface characterization using ellipsometry, water contact angle measurements and X-ray photoelectron spectroscopy (XPS), demonstrated the success of NAC immobilization at 4 mg/mL. Quartz crystal microbalance with dissipation (QCM-D) demonstrated that surface immobilized NAC decreases protein adsorption to Ch coatings. Biological studies confirmed that immobilized NAC4 avoids methicillin-resistant *Staphylococcus aureus* adhesion to Ch coating, impairing biofilm formation, without inducing cytotoxic effects. This is particularly interesting towards further developments as a prevention coating.

## Introduction

N-acetylcysteine (NAC) is a known drug widely applied in a number of different clinical conditions ranging from chronic bronchitis, acetaminophen overdose treatment, chemotherapy-induced toxicity treatment, to HIV/AIDS and psychiatric disorders^1^. Adding to this broad activity, NAC has been reported as having an excellent safety profile^2^. NAC has been researched for further applications such as tissue regeneration, mostly in orthopedic and dental implants, to reduce cytotoxicity and improve osteointegration^3–5^. Interestingly, NAC has also antimicrobial properties against both Gram-positive and Gram-negative bacteria^6–10^. Although NAC antimicrobial mechanism is not fully described, it is known that the sulfhydryl moiety of NAC acts as an antioxidant, playing a role in free radical scavenging and therefore, helping the immune system in destroying intermolecular or intramolecular disulfide bonds of bacterial proteins^1,11,12^. Additionally, an antibiofilm activity has been reported, including reduction of bacteria adhesion, reduction of extracellular polysaccharide production and disruption of mature biofilms^6,8,13^. Therefore, NAC appears to be a promising preventive or even coadjuvant in the treatment of infections, which has prompted its research after being physically adsorbed onto collagen scaffolds^12^, and its patenting as a catheter lock solution^14^. Although having variable success rates, these strategies stressed the importance of a high long lasting local NAC concentration, in order to achieve better performance. Taking these findings in consideration we hypothesized the application of covalently immobilized NAC onto a polymer in order to develop an antimicrobial coating to avoid implant-related osteomyelitis infection. To that end, we chose chitosan, a natural cationic polysaccharide with recognized antimicrobial^15–18^ and osteoconductive properties^19–21^. Chitosan can be easily functionalized, and a number of chitosan derivatives have been studied for pharmaceutical and medical applications^22–25^. Particularly, thiolated chitosans have been explored for their potential as drug delivery systems, as they possess improved mucoadhesive and permeation enhancing properties^25,26^. Moreover, their strong cohesive properties make them highly suitable excipients for controlled drug release^25,27^. Recently, Fernandes *et al*.^28^ published a study where different soluble thiolated chitosans had their antimicrobial activity reported both with a bacteria membrane model and with bacteria. In all these studies chitosan functionalization was performed in solution in the presence of excess NAC^25,27,28^. However, as this approach does not guarantee –SH exposure to the surface and may promote unintended crosslinking^24^, we propose NAC-functionalization on pre-formed chitosan films to avoid bacterial adhesion, without impairing osteoblast proliferation. For that, we optimized the production of chitosan films with increasing NAC concentrations, assessed film stability and evaluated their biological impact on: (i) Methicillin-resistant *Staphylococcus aureus* (MRSA) adherence, proliferation and biofilm production, and (ii) Osteoblast MC3T3-E1 cell line metabolic activity and morphology.

## Results

### Optimization of NAC immobilization

Control film (Ch_Buffer), Ch incubated with carbodiimide reagents EDC and NHS (Ch_EDC) and Ch functionalized with NAC at concentrations ranging from 0.4 (Ch_NAC0.4) to 20 mg/mL (Ch_NAC20) were analyzed by XPS, ellipsometry, water contact angle measurements and IRRAS.

#### X-ray Photoelectron Spectroscopy (XPS)

**Figure 1:**
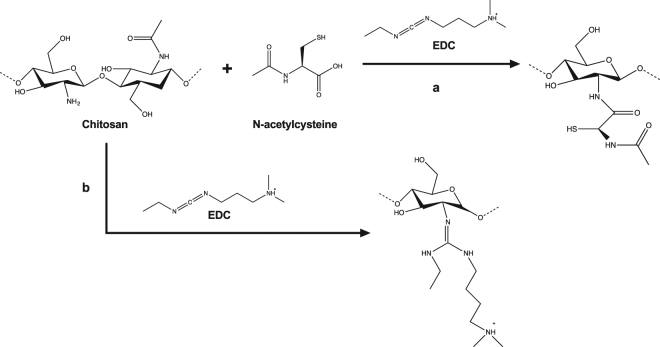
(**a**) Chitosan modification with NAC through carbodiimide chemistry. (**b**) Chitosan modification through carbodiimide chemistry in the absence of NAC.

XPS survey spectra demonstrated the absence of surface contaminants, since no other elements than those expected were detected (data available online in supplementary data SSD Fig. 1). The relative atomic composition of all surfaces is described at Table 1.

**Table 1:** Surface atomic composition (%) calculated from high-resolution XPS spectra of different chitosan samples.

Samples	Atomic % (All Elements)							
	*Au4f*	*C1s*	*N1s*	*O1s*	*S2p*	Atomic % (*S2p*)		
						~162 eV S-Au	164–165 eV S-H	168–169 eV SO_3_
Ch_Buffer	0.1	67.3	7.6	24.9	0.2	0	0	100
Ch_EDC	ND	66.9	12.4	20.7	ND	0	0	0
Ch_NAC0.4	0.4	64.5	12.3	22.8	ND	0	0	0
Ch_NAC2	0.3	64.6	12.7	22.1	0.3	0	100	0
Ch_NAC4	0.2	65.9	12.0	21.5	0.4	0	100	0
Ch_NAC8	0.6	63.9	11.5	23.5	0.4	0	100	0
Ch_NAC12	1.6	62.8	10.7	24.0	0.9	47	53	0
Ch_NAC16	7.7	60.8	10.4	19.0	2.1	58	42	0
Ch_NAC20	3.6	55.5	10.6	29.2	1.2	58	42	0

Ch_Buffer was in accordance with our previous reports^29,30^. As expected, a small excess of C1s atomic percentage in comparison to the theoretical value was observed (supplementary SSD Table 1), due to the common carbon contamination that happens when samples are not preserved in high vacuum conditions^29^. Consequently, the relative atomic percentage of *N1s* and *O1s* was slightly lower than expected. Moreover, the presence of *S2p at* very low percentage can be assigned to oxidized sulfur (*~168 eV)* from MES buffer.

XPS results also demonstrated that carbodiimide reaction in the absence of NAC, allowed the direct immobilization of EDC to the Ch backbone (as depicted in Fig. 1b) as previously suggested by us^17^.

**Figure 2:**
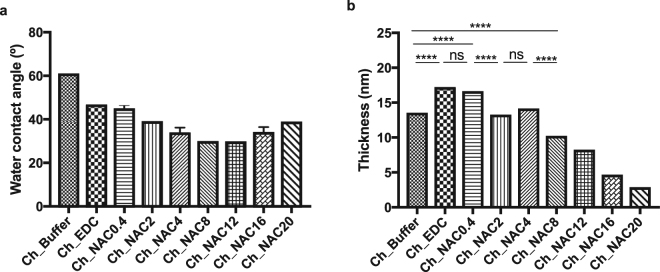
(**a**) Water contact angle measurements of Ch and Ch-modified films. Statistical analysis performed with non-parametric Kruskal-Wallis test, no statistical differences were found; Data represent mean ± Standard deviation. (**b**) Ellipsometry measurements of Ch and Ch-modified films. ****Statistically different (p < 0.0001) (one-way ANOVA test). Data represent mean ± Standard deviation.

Ch_EDC shows a clear increase of *N1s* and a decrease of *O1s*, which is congruent with expected values (supplementary Table 1). Regarding *C1s*, Ch_EDC has similar total carbon amounts comparing to Ch_Buffer, however high-resolution *C1s* spectra allowed the detection of a higher contribution in the 287.9 eV peak assigned to C=N, suggesting the EDC immobilization onto Ch_Buffer (SSD Fig. 2).

Since EDC also has nitrogen, NAC immobilization can be specifically followed by the appearance of S2p that increased with NAC concentration present at the reaction. However, the high increase of the relative Au percentage on samples prepared with NAC concentrations >= 8 mg/mL suggests the partial detachment of the chitosan films. It is well established that free thiol groups have high affinity towards gold, therefore a NAC concentration threshold above Ch_NAC8 could be responsible for chitosan replacement by direct immobilization of NAC on gold. This explanation is supported by the observation of a ~162 eV peak in samples Ch_NAC12 to Ch_NAC20, assigned to S-Au bond.

Nevertheless, the presence of S2p at 164-165 eV (-SH), the small decrease of Au and small increase of O, suggest NAC immobilization onto Ch films until 4 mg/mL although some EDC could be present in lower NAC concentrations.

#### Water optical contact angle (WCA) analysis

Water optical contact angles of the control and NAC-modified Ch films are presented on Fig. 2a.

Ch_EDC sample presented increased hydrophilicity, as compared to unmodified Ch, which is congruent with the EDC immobilization onto chitosan as proposed at Fig. 1b. Chitosan functionalization with growing NAC concentrations, resulted on increasing hydrophilicity, as the water contact angle decreased from 61 ± 3° to minimum of 30 ± 1° at Ch_NAC12. Beyond this NAC concentration, water contact angle increased, which agrees with the aforementioned hypothesis of NAC-induced detachment of the hydrophilic chitosan films from gold, in consequence of competing direct thiol-mediated NAC binding to the gold surface above a NAC threshold concentration.

#### Ellipsometry

**Figure 3:**
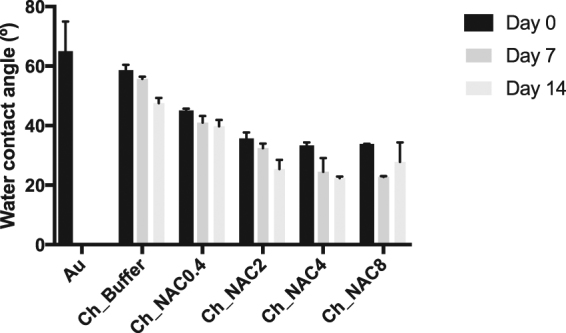
Water contact angle measurements over time. Statistical analysis performed with non-parametric Kruskal-Wallis test. No statistical differences were found; Data represent mean ± Standard deviation.

The spin coating process resulted in uniformly distributed Ch with 14 ± 0.6 nm thickness, which remained stable even after the reaction protocols used, as there were no thickness changes between freshly made films and buffer-incubated films (see supplementary data SSD Fig. 3). Figure 2b presents thickness of Ch before and after surface modification with NAC concentrations, ranging from 0.4 to 20 mg/mL.

The Ch_Buffer thickness was augmented after EDC reaction to 17 ± 0.9 nm, as formerly proposed by the chemical pathway presented in Fig. 1b. NAC functionalization with increasing NAC concentrations resulted on sequential thickness reduction, which may be explained by EDC replacement for NAC. Notwithstanding, thickness decrease reaches a threshold at Ch_NAC4, as beyond this concentration films are thinner than control Ch_Buffer. These results are in accordance with the increase of Au relative percentage (Table 1), reinforcing the suggestion that direct immobilization of NAC onto gold occurs with partial detachment of Ch films.

#### Infrared reflection absorption spectroscopy (IRRAS)

**Figure 4:**
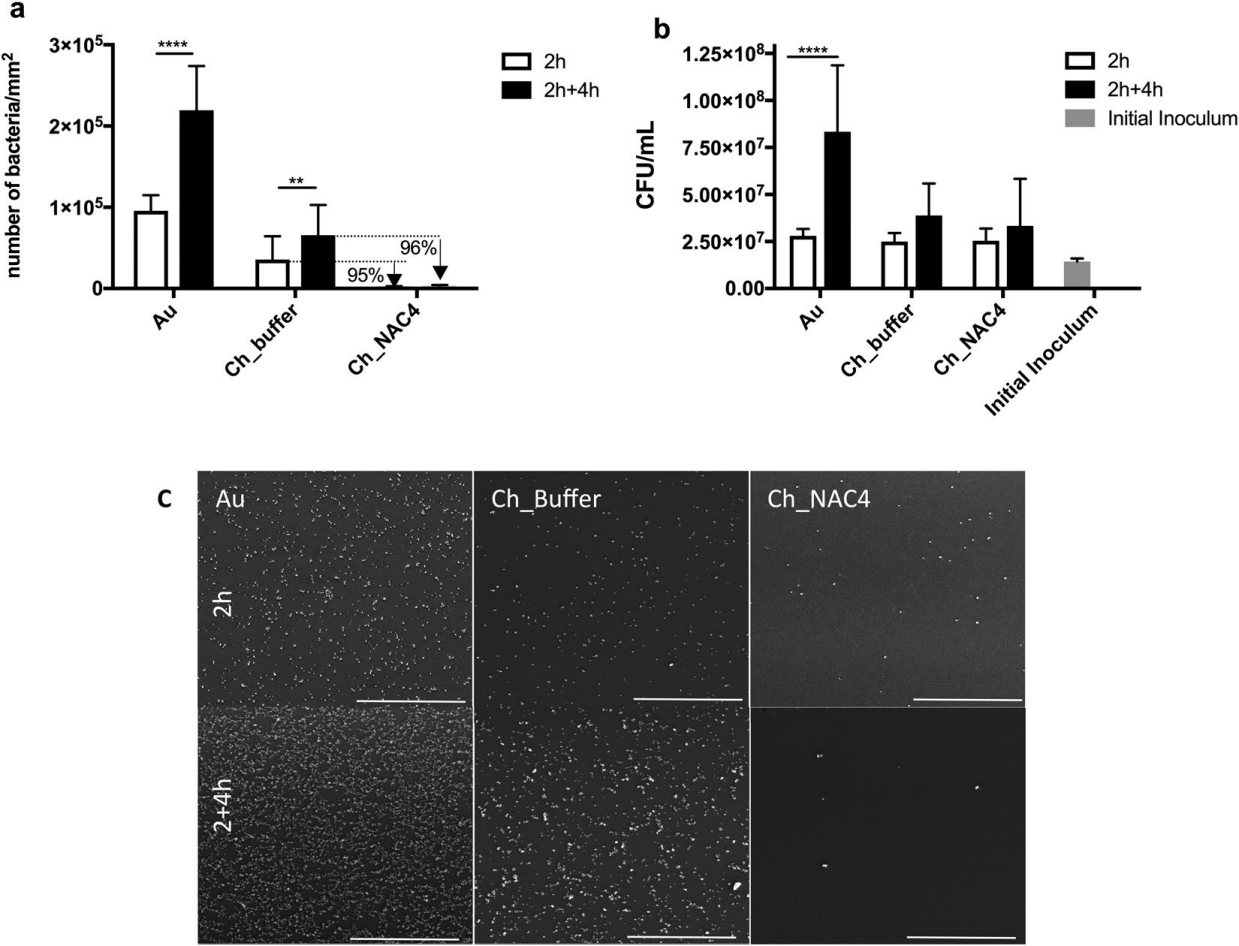
(**a**) *S. aureus* adhesion on Au, Ch and NAC-modified Ch films after 2 h incubation in MHB (white bars) and 4 h re-incubation on fresh medium MHB after the 2 h pre-incubation period (black bars) Data represent mean ± Standard deviation. (**b**) *S. aureus* CFU counts of the supernatants after 2 h incubation period (white bars) and after 4 h re-incubation in fresh medium (black bars) (one-way ANOVA **p < 0.003; ****p < 0.0001); Data represent mean ± Standard deviation. (**c**) SEM images of *S. aureus* adherent to Ch and NAC-modified Ch films after 2 h and 2 + 4 h incubation periods. Au surfaces were used as controls. Scale bar: 100 µm.

IRRAS analyses of chitosan thin films, before and after immersion on buffer solution, showed that the films remained stable even after chemical procedures undertaken (data not shown). IRRAS spectra of control and NAC-modified chitosan up to 8 mg/mL can be found online in supplementary data SSD Fig. 4.

Ch_Buffer spectrum allowed the identification of characteristic absorption bands of chitosan, in agreement with previous literature^29–33^.

Both NAC-modified chitosan and EDC-modified chitosan spectra show an increase of the amide I absorption band (1654 cm^−1^), which can be assigned to the amide bond established between free amine groups of Ch and carboxylic groups of NAC, and to an imine intermediate (C=N stretching typically ranges from 1690 to 1630 cm^−1^), respectively^34^. No difference was observed among spectra of Ch modified with different NAC concentrations.

#### Functionalization stability

To determine whether NAC covalently bound to Ch (concentrations below 8 mg/mL), remained stable over time, a soak test was performed in PBS for 14 days at room temperature. Results are depicted on Fig. 3.

As depicted in Fig. 3 samples presented a tendency to higher hydrophilicity over time, which is not statistically significant. If film degradation had occurred, part of the gold substrate should be exposed resulting on a higher water contact angle. None of the samples had water contact angle augmentation (with the exception of Ch_NAC8 at day 14), the opposite occurred.

**Figure 5:**
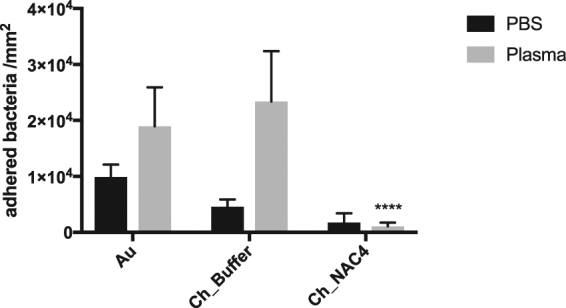
*S. aureus* adhesion on Au, Ch_Buffer and Ch_NAC4 after 2 h incubation in PBS or PBS supplemented with 1% human plasma; Statistical analysis performed with non-parametric Kruskal-Wallis test ****p < 0.0001. Data represent mean ± Standard deviation.

**Figure 6:**
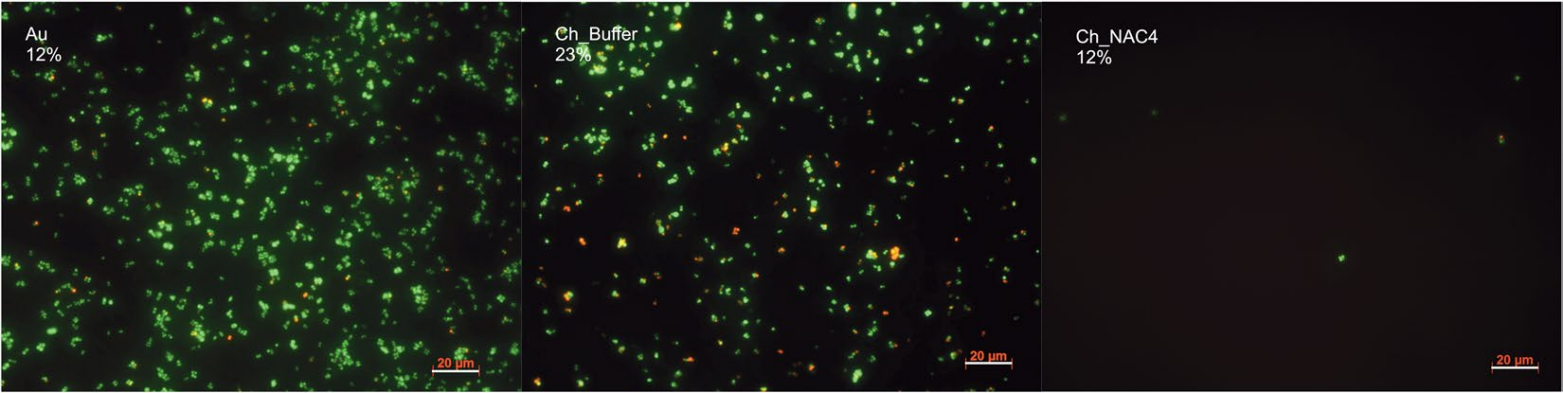
Fluorescent microscopy images of *S. aureus* adherent to Ch and NAC-modified Ch films after 2 h incubation period and stained with LIVE/DEAD^®^ Bacterial Viability Kit (Baclight^TM^). Bacteria stained in green are alive and bacteria stained in red are dead. Au surfaces were used as controls. Scale bar: 20 µm. The percentages presented correspond to % of Dead bacteria.

Further stability assessment was performed by a second soak test in PBS but incubation was performed at 37 °C for 7 days. Surfaces were then analyzed by XPS and their XPS relative atomic composition, at day 0 and day 7, are presented in Table 2. Moreover, XPS spectra surveys can be found in supplementary data SSD Fig. 5 and S2p high-resolution spectra in SSD Fig. 6.

**Table 2:** Surface atomic composition (%) calculated from survey XPS spectra of different chitosan samples before and after immersion in PBS at 37 °C for 7 days.

Samples						
Elements	Ch_Buffer		Ch_NAC4		Ch_NAC8	
	**Day 0**	**Day 7**	**Day 0**	**Day 7**	**Day 0**	**Day 7**
C1s	67.7	64.4	66.3	64.1	64.7	61.6
N1s	5.0	8.3	10.7	12.3	11.5	13.3
O1s	26.9	27.3	22.6	23.4	23.2	24.8
S2p	0.3	0.0	0.44	0.24	0.54	0.33

As described above (Table 1), a small oxidized S2p from MES buffer was observed on Ch_Buffer (day 0) that disappeared after 7 days in PBS at 37 °C. The decrease of C1s and increase of N1s on Ch_Buffer sample after 7 days can be explained by the release of carbon contamination, which is always observed on chitosan that is not maintained in vacuum conditions, since values are closer to the expected (theoretical values SSD_Table 1). This fact can also explain the increase of N1s and the decrease of C1s in both Ch_NAC4 and Ch_NAC8. Although a small decrease in the relative % of S2p was observed on survey measurements of NAC surfaces, their absolute amount was not so different when the S2p XPS high resolution spectrum was analyzed (SSD_Fig. 6), particularly for Ch_NAC4. Moreover, no oxidation of S2p was detected (SSD_Fig. 6). These results demonstrate the presence of the coating at the end of the 7 days period (PBS and 37 °C).

The evidences gathered throughout different surface characterization techniques suggested that 4 mg/mL is enough to reach a stable chitosan film containing NAC covalently immobilized. Therefore, in order to evaluate the effect of covalent immobilization on the antimicrobial properties of NAC, all biological assays were performed using this NAC concentration (Ch_NAC4).

### Antimicrobial Activity

The antimicrobial properties of NAC-functionalized Ch were evaluated by the assessment of their capacity to avoid *S. aureus* adhesion, proliferation and biofilm formation, as this is the most prevalent species in osteomyelitis^35,36^.

#### Adhesion assays

The effect of developed thin films on methicillin-resistant *S. aureus* adhesion is demonstrated on Fig. 4a.

As seen on Fig. 4a, after the 2 h incubation period (white bars), bacteria adhered preferably to the Au substrates used as control. Ch film coating promoted a ~76% decrease in bacterial adhesion compared to Au, as previously described by us^17^. Covalent immobilization of NAC clearly decreased bacterial adhesion by ~95% regarding Ch films, demonstrating the anti-adhesive capacity of Ch_NAC4 films. This anti-adhesive behavior of Ch_NAC4 is not compromised in the presence of human plasma proteins as depicted on Fig. 5.

Figure 4b represents bacterial behavior at the supernatants, which shows that bulk phase bacteria were able to duplicate in number during the 2 h incubation period in MHB (white bars), as compared to the initial inoculum (grey bar). No significant difference was found between the amount of bacteria in different samples. This can be explained by the high initial inoculum that was free to proliferate on this bulk phase.

#### Proliferation assays

To assess the degree of proliferation of surface adherent bacteria in the 2 h incubation assay (white bars on Fig. 4a), surfaces were re-incubated in fresh MHB medium for 4 h. Results obtained are depicted by the black bars on Fig. 4a and b.

Adherent bacteria on Au and Ch_Buffer surfaces were able to proliferate, since the number of surface adherent bacteria duplicated regarding the respective values obtained on the previous 2 h adherence assay (white bars). Also, at supernatants level (black bars on Fig. 4b), high colony-forming units (CFUs) were found, suggesting that bacteria were further able to detach and proliferate in bulk phase.

In contrast, surfaces with covalently immobilized NAC maintained the low amount of adherent bacteria throughout the extended 4 h incubation period, demonstrating that adherent bacteria could not proliferate on the surface (black bars on Fig. 4a). In addition, although some adherent bacteria could be detached from the surface and proliferate in solution (black bars on Fig. 4b), they were not able to adhere on NAC-surfaces since, as described above, the number of adherent bacteria did not increase on these surfaces. Such anti-adhesive effect is further reinforced by visualization of adherent bacteria on the different surfaces by SEM, as depicted on Fig. 4C. SEM images correlate with findings made on the adhesion assays (Fig. 4a).

#### Bacterial Viability in adhesion assay

Viability of bacteria adhered to the surfaces was assessed using LIVE/DEAD^®^ Bacterial Viability Kit (Baclight^TM^). The kit contains two fluorescent dyes: Syto9, which stains all bacteria in green; and propidium iodide (PI) that can only crossover damaged bacteria membranes and gives red stained bacteria. As PI quenches the fluorescent emission of Syto9, it is assumed that green cells are alive whereas red cells are dead. Figure 6 depicts representative images of Au, Ch_Buffer and Ch_NAC4 after a 2 h incubation adhesion assay.

Ch_Buffer presented 23% of dead bacteria adhered to the surface. This result was expected, since we previously demonstrated the antimicrobial properties of chitosan ultrathin films^37^. Nevertheless, the low number of adherent bacteria found on Ch_NAC4 corresponds to living bacteria, demonstrating that this surface is exerting anti-adhesive properties rather than bactericidal properties.

#### Quartz Crystal Microbalance with Dissipation (QCM-D)

**Figure 7:**
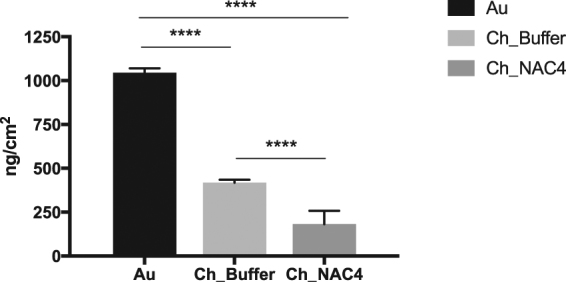
Mass of proteins from 1% (v/v) human plasma adsorbed on Au, Ch_Buffer and Ch_NAC4 surfaces (Voigt model). Data represent mean ± Standard deviation (****p < 0.0001, One-Way Anova).

In order to clarify if the Ch_NAC4 anti-adhesive performance could be related with non-fouling properties and since the presence of proteins did not affect bacterial adhesion (Fig. 5), a QCM-D assay was used to evaluate human blood plasma proteins adsorption on the surfaces. Results are presented in Fig. 7.

QCM-D is able to detect mass increase (Δm) and structural changes such as swelling, due to the simultaneous recording of frequency (Δf) and dissipation (ΔD). Figure 7 shows the total amount of human blood plasma proteins adsorbed on different surfaces calculated using the Voigt model after 2 h of incubation followed by 2 h of rinsing with PBS. Voigt viscoelastic modeling was used to perform the QCM-D data fitting, since this model takes in account the energy losses in the system (i.e dissipation), correcting the deviation introduced by this factor. Protein adsorption was observed in all surfaces, although the mass of adsorbed proteins was lower on Ch_NAC4 (≈180 ng/cm^2^) than on other surfaces (Ch_Buffer ≈ 400 ng/cm^2^ and Au ≈ 1000 ng/cm^2^).

#### Biofilm assay

**Figure 8:**
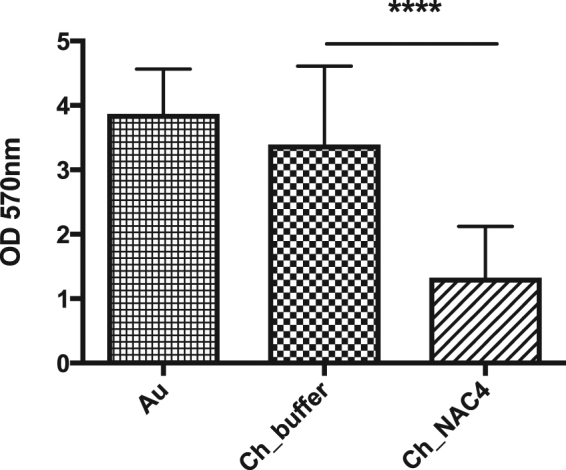
Effect of Ch and NAC-modified Ch surfaces on *S. aureus* total biofilm biomass formation. Statistical analysis performed with non-parametric Kruskal-Wallis test ****p < 0.0001; Data represent mean ± Standard deviation.

In order to evaluate surface activity during long-term bacteria interaction, biofilm assays were performed. Crystal violet (CV) staining was used as an indicator of total biofilm biomass. Results, presented in Fig. 8, clearly demonstrate that covalent immobilization of NAC provides anti-biofilm properties to the chitosan thin film. Ch_NAC4 presented the lowest value of biofilm biomass that was ~3-fold lower than that of control Ch_Buffer. No difference was observed between the controls Au and Ch_Buffer.

### Influence of NAC-modified surfaces on osteoblastic cells

In order to evaluate possible cytotoxic effects of the modified surfaces on cell interaction/adhesion, osteoblastic MC3T3-E1 cells (a pre-osteoblast cell line) were cultured on top of Ch and Ch_NAC4 surfaces for 14 days under osteogenic conditions. Cell metabolic activity, cell adhesion, integrity and morphology were assessed at days 3, 7 and 14 of culture.

**Figure 9:**
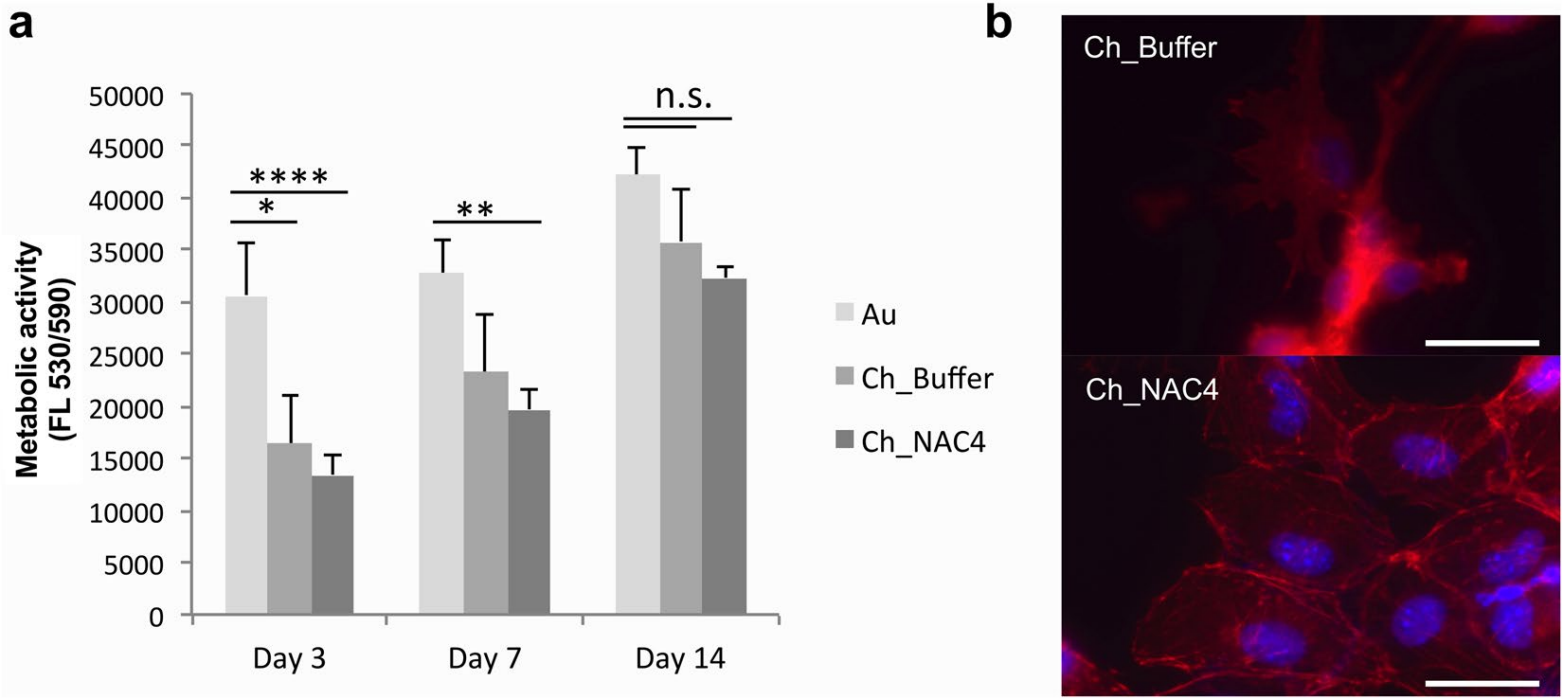
(**a**) MC3T3-E1 cell metabolic activity after 3, 7 and 14 days in contact with Au, Ch and Ch_NAC modified surfaces. Data represent mean ± Standard deviation (n = 6). (non-parametric Kruskal-wallis analysis *p < 0.01; **p < 0.003; ****p < 0.0001; n.s.- non-significant). (**b**) Osteoblast morphology following 14 days of culture on top of Ch and Ch_NAC4 modified surfaces. Scale bar 50 μm. The images are representative of three independent experiments. F-actin filaments are stained in red and nuclei are stained in blue.

Regarding MC3T3-E1 proliferation, cells became confluent on Au control sample at day 3 and continued to proliferate on the followings days, covering the whole surface at day 14 of culture. On Ch_Buffer, MC3T3-E1 cells tended to agglomerate and grow forming big clusters spread throughout the Ch film surface, as previously described for other osteoblast-like cells^38^. On the Ch_NAC4 sample, a low number of adhered cells were observed at the initial time point (day 3), and cells were dispersed but elongated. As time progressed, cells were able to proliferate to cover most of the sample surface at day 14, as shown in Fig. 9b. In all cases, cells were well spread with a typical osteoblast-like morphology.

Therefore, as depicted in Fig. 9a, Ch film was responsible for a reduced MC3T3-E1 metabolic activity regarding Au control surface at early time point (Day 3), which was overcome on latter time points (Day 14), where metabolic activity is similar to Au control. Importantly, the metabolic activity differential between Ch_NAC4 and Ch_Buffer control was never superior to 30%, demonstrating that NAC-modified surfaces have no cytotoxic potential, according to ISO 10993-5:2009(E). These data suggest that MC3T3-E1 cells plated on top of NAC surfaces were viable and able to proliferate along the time of culture.

## Discussion

NAC is an FDA-approved drug clinically applied on a broad range of pathologies^1^. Moreover, NAC has been reported to have antimicrobial activity against a variety of microorganisms, including the clinically relevant MRSA^6,13,39,40^. However, since NAC serum levels obtained with intravenous infusions (about 0.035 mg/mL)^41^ are much lower than the antimicrobial concentration reported in most studies (between 4 and 80 mg/mL)^13,40,42,43^, strategies that accomplish high local concentrations would be beneficial^10,14^.

In this study, covalent immobilization of NAC was performed, in order to obtain long-lasting high local concentration of the drug onto a chitosan-derived implant-related coating. For the production of NAC-functionalized chitosan films, water-based carbodiimide chemistry was applied to avoid the use of toxic organic solvents. In this frequently used chemistry, carbodiimide (EDC) reacts with the carboxylic acid from NAC to produce the O-acylisourea intermediate with an activated leaving group that can be stabilized upon reaction with NHS, producing a yet reactive intermediate. This intermediate will further react with primary amines from Ch to form amide bonds^24,28^. Differently from the literature, our strategy was to immobilize NAC onto the surface of previously prepared Ch films, to ensure full exposure of NAC. However, applying the aforementioned immobilization chemistry on a pre-formed chitosan film poses some challenges, namely a strict control of pH so that, on the one hand, the reaction is efficient and, on the other, solubilization of the chitosan film is avoided (while chitosan films solubilize at pH below ~6.5^23^, literature reports suggest pH~5 as optimal for carbodiimide chemistry^44^). Also, a limited number of surface Ch free amines are available (Ch degree of acetylation (DA) ~15%) which might explain the NAC saturation point at 4 mg/mL. Noteworthy, a two-step immobilization procedure was tested, namely (i) NAC activation with EDC, and (ii) subsequent incubation with Ch film, but immobilization yield was not improved (data not shown).

The production of Ch-modified films was optimized and characterized by a number of different techniques (XPS, ellipsometry, water contact angle measurements, and IRRAS). Strong evidence on successful NAC covalent tethering was brought by the sum of different contributions. The success of NAC immobilization was demonstrated by XPS through observation of the S2p (~164 eV) peak assigned to free -SH group for NAC concentrations up to 8 mg/mL. However, the increased intensity of the Au4f peak (XPS) and the decreased film thickness (ellipsometry) for samples prepared with 8 mg/mL, and above, suggested a maximum NAC immobilization threshold at 4 mg/mL, corresponding to a 8.8% derivatization degree. Above this concentration threshold, some Ch detachment occurs due to the NAC’s affinity to gold, which is supported by XPS observation of the Sp2 peak at ~162 eV (Au-S).

We also demonstrated that, for low NAC concentrations, some EDC immobilization may occur, as suggested in Ch_EDC by the increase of its i) amide I absorption band (IRRAS), ii) C1s and N1s atomic percentages (XPS), iii) film thickness (ellipsometry), and iv) hydrophilicity (WCA measurements), when compared to Ch_Buffer.

Regarding stability of NAC functionalization, Ch_NAC4 was stable for 7 days in PBS at 37 °C, as the overall relative atomic composition obtained by XPS suggests Ch_NAC4 film maintenance at the end of the incubation period, with minimal NAC leaching. Also, by water contact angle measurements no significant hydrophobic increase was observed on surface wettability. These results differ from those reported by Harris *et al*.^45^, where NAC covalently immobilized onto poly(dimethyl)siloxane presented low yield and stability. This might be explained by the well-known high stability of amide bonds, as those formed between primary amines of chitosan and carboxyl groups of NAC, herein reported, in opposition to the ester bonds established between poly(dimethyl)siloxane hydroxyls and NAC carboxyl groups.

Regarding bacterial adhesion assays, surfaces were challenged with a very high initial inoculum over 2 h, to assess the Ch_NAC response to harsh conditions. As already reported in some studies, chitosan films were able to reduce bacterial adhesion of about 76%, as compared to naked Au surfaces^17,37^. This anti-adhesive property was dramatically increased for Ch_NAC4, by 95% and ~98% as compared to Ch and Au surfaces, respectively, and was not abolished in the presence of plasma proteins. The observed anti-adhesive properties of Ch_NAC4 may result from the superficial interactions between bacterial cell surface and the immobilized NAC coating. Such anti-adhesive properties might be simply ascribed to physical-chemical interactions, namely Ch_NAC4 low water contact angle, as less hydrophobic surfaces have been correlated with lower protein adsorption and bacterial adhesion^46^. Indeed, QCM-D assay revealed that protein adsorption was lower on Ch_NAC4 than on Ch_Buffer and Au. However, some protein adsorption was observed on Ch_NAC4 and thus, the lower bacterial adhesion may be related not only with the amount but also with the type/conformation of the adsorbed proteins^47^. It is well known that non-adhesive proteins such as albumin avoid bacterial adhesion, namely *S. aureus* ^48^. Moreover, Linnes *et al*.^47^ also demonstrated that fibronectin coatings were able to decrease *S. epidermidis* adhesion to biomaterials.

On proliferation assay, the capacity of surface adherent bacteria (from the 2 h adhesion assay) to proliferate/detach from the surface was evaluated. Results demonstrated that NAC-functionalized samples had no increased number of adhered bacteria after incubation in a fresh medium. However, some bacteria were found in the supernatants demonstrated that a minimum number of adherent bacteria were able to detach from the surface and proliferate in solution (Fig. 4a and b). Importantly, the anti-adhesive effect of the NAC-functionalized surface clearly avoids adhesion of planktonic bacteria to the surface, since the number of attached bacteria did not increase on this surface.

Bacterial viability assay clearly demonstrated that NAC effect is not bactericidal but anti-adhesive. Therefore, the antimicrobial properties attributed to soluble NAC, such as NAC-mediated destruction of disulfide bonds in proteins^11^, production impairment of extracellular polymeric substances (EPS) which are precursors of biofilm matrix^6,42,49^, may not be the explanation for immobilized NAC (Ch_NAC4).

The anti-adhesive effect observed in Ch_NAC4 may be explained by a combination of factors, namely i) the reduction of free available amine groups of Ch, which are related with the adherent dead bacteria observed on Ch_Buffer; ii) the increase of surface hydrophilicity, which can decrease bacterial adhesion directly or indirectly through the decrease of protein adsorption or the control on the type and conformation of adsorbed proteins. Therefore, it seems that the high local concentration of immobilized NAC is attributing surface characteristics that although interesting from a bacterial adhesion prevention point of view, is not congruent with the literature proposed mechanism of action. Nevertheless, it remains to be clarified if the exposed thiol group is having any direct contribution in preventing specific adhesion (e.g. degrading relevant disulfide bridges of bacterial adhesins), or if the overall mechanism is purely non-specific anti-adhesive.

Ch_NAC4 anti-adherence properties were further highlighted through the biofilm total biomass quantification, as this modified surface efficiently reduced the amount of biofilm. In contrast, the Ch_Buffer surface had its antimicrobial activity overwhelmed by the extended incubation period, meaning that the initial bactericidal effect of the surface was not sufficient to withstand the higher bacterial adhesion allowed by this surface. As this surface does not prevent protein adsorption, bacteria (both dead and alive) accumulate over time, ending up by presenting a biofilm total biomass similar to the one found for the control Au sample. The Ch_NAC4 anti-biofilm activity can be correlated with its anti-adhesion properties.

Effects of soluble NAC on eukaryotic cells have been previously addressed, with NAC being described as non-cytotoxic and even cell stimulating drug^3,5,50,51^. Since our most promising antimicrobial surface, Ch_NAC4, is hydrophilic and hinders bacteria adhesion, it would have been reasonable to assume that it might lead to poor cell adhesion and proliferation^45^. However, as NAC was immobilized on chitosan known for its osteogenic properties^19–21,52^, we expected Ch_NAC4 to present suitable cell adhesion and proliferation properties. Our results showed that the metabolic activity of cells adhered to NAC-modified samples was similar to those of controls (Au and Ch_Buffer), exhibiting a normal osteoblastic morphology. As such, Ch_NAC4 surfaces did not induced cytotoxic effects.

## Conclusion

A bacterial anti-adhesive coating was developed by functionalizing a pre-formed chitosan film with NAC. This was achieved by employing a water-based carbodiimide chemistry (free of toxic organic solvents), and careful tuning of NAC concentration. Surface characterization using several techniques demonstrated the success of NAC immobilization at 4 mg/mL. Biological studies confirmed Ch_NAC4 to be a promising material, as it avoids bacterial adhesion, and impairs biofilm formation, without inducing cytotoxic effects. This is particularly interesting towards further developments as a prevention coating.

## Materials and Methods

### Preparation of Chitosan Ultrathin Films

A re-precipitation method^31^ was used to purify commercial squid pen chitosan (France Chitine), resulting on a molecular weight of 283–472 kDa with a degree of acetylation (DA) of ~15%. Chitosan ultrathin films (Ch) were prepared as previously described by us^17,30^ by spincoating (9000 rpm, 1 min) chitosan solution (0.4% in acetic acid w/v) onto gold substrates (Au) (1 × 1 cm^2^). Ch was neutralized (0.1 M NaOH), rinsed with type I water and dried with a gentle stream of argon. Au, produced as previously described by us^53^, was chosen due to its higher suitability for some of the surface characterization techniques used.

### Functionalization with NAC

Functionalization of Ch with NAC was optimized using different NAC concentrations, namely 0.4, 2, 4, 8, 12, 16 and 20 mg/mL. Ch films were treated with a solution of 0.2 M 1-Ethyl-3-[3-dimethylaminopropyl] carbodiimide hydrochloride) (EDC; Sigma-Aldrich), 0.05 M N-hydroxysulfosuccimide (NHS; Sigma-Aldrich) and different NAC concentrations (Merck), in 0.1 M (N-morpholino)ethanesulfonic acid (MES; Sigma-Aldrich) buffer at pH 6.5, for 1 h, at 37 °C and 100 rpm. The modified films were rinsed (type I water) in an ultrasound bath (1 min; Bandelin Sonorex Digitec Bath 35 kHz) and then rinsed twice (type I water).

### Surface Characterization

Firstly, samples were dried on a Vacuum Oven (Raypa, EV 50) for 1 h at 40 °C.

#### X-ray Photoelectron Spectroscopy (XPS)

XPS measurements were performed using a Kratos Axis Ultra HSA (UK) with PISCES software for data acquisition and analysis (from CEMUP – Centro de Materiais da Universidade do Porto). The analysis was carried out with a monochromatic Al Kα X-ray source (1486.7 eV), operating at 15 kV (90 W), in FAT mode (Fixed Analyser Transmission). Survey spectra over a range of 0–1150 eV were collected with analyzer pass energy of 80 eV. High resolution C1s, O1s, N1s, S2p and Au4f spectra were collected with analyzer pass energy of 40 eV. The photoelectrons were analyzed at a take-off angle of 70°. The binding energy (BE) scales were referenced by setting the C1s BE to 285.0 eV. Data acquisition was performed with a pressure lower than 1 × 10^−6^ Pa, and it was used a charge neutralization system. All spectra were fitted using XPS peak fitting software (XPSPEAK Version 4.1). All carbon spectra were fitted using asymmetrical 70% Gaussian/30% Lorentzian profiles.

#### Water Contact Angle (WCA)

Contact angle measuring system from Data Physics, model OCA 15, equipped with a video CCD-camera and SCA 20 software was used with sessile drop method, as described at^53^. Drops of type I water (4 µL) were deposited onto the samples surfaces and images were taken every 2 s over 300 s. Droplet profiles were fitted using Young-Laplace formula, and the WCA of each sample was calculated by extrapolating the time dependent curve to zero. Results are the average of two measurements on three independent samples.

#### Ellipsometry

Film thickness was measured with an imaging ellipsometer, model EP3, from Nanofilm Surface Analysis, in a polarizer-compensator-sample-analyzer (PCSA) mode (null ellipsometry). The light source was a solid-state laser (532 nm). The Au refractive index (n) and extinction coefficient (k) were determined by using a delta and psi spectrum with a variation of angle between 65° and 71° [(n) of 0.6477 (k) of 2.6007 was obtained for Au]. Four regions-of-interest were used to correct for any instrument misalignment. The thickness of modified Ch was determined considering n = 1.54 and k = 0, for the Ch^54^. Results presented are the average of three measurements on each of three samples.

#### Infrared reflection absorption spectroscopy (IRRAS)

IRRAS spectra were obtained on a Perkin Elmer FTIR spectrophotometer, model 2000, coupled with a VeeMax II Accessory (PIKE) and a liquid-nitrogen-cooled MTC detector. Instrument purge with dry nitrogen (for 5 min before and during each sample analysis) was performed to avoid water vapor adsorption. Similar Au was used as a background. Incident light was p-polarised and spectra were collected using the 80° grazing angle reflection mode. For each sample, 100 scans were performed with 4 cm^−1^ resolution.

#### Functionalization stability

Stability of immobilized NAC assay was performed by soaking NAC-modified films in PBS for 7 and/or 14 days at 37 °C and room temperature, respectively. Samples representative of Day 0 (controls) were immersed in PBS for 1 h and then rinsed and dried under a gentle stream of argon. Surfaces from the 7 days soaking period at 37 °C were analyzed by XPS as explained in section 5.3.1. Surfaces from the 14 days soaking period at room temperature had their WCA measured before (Day 0) and after 7 and 14 days soaking. Samples were rinsed as explained at 2.2. Results are the average of two measurements on three independent replicates.

### Bacterial assays

#### Bacterial Strains, Media and Growth Conditions

Methicillin-resistant *Staphylococcus aureus* strain (MRSA) was bought from the American Type Culture Collection (ATCC 33591), and streaked onto Tryptic Soy Agar (TSA; Merck). Bacterial suspension was obtained from overnight culture in Tryptic Soy Broth (TSB; Merck) at 37 °C, 150 rpm, and concentration was adjusted by Optical Density (600 nm) (confirmed by retrospective viable count).

#### Surfaces disinfection

Test surfaces were incubated successively in 70% ethanol and sterile water, and then dried in sterile environment. These samples were then placed on 24-well flat bottom cellular suspension plates (Sarstedt, Ldt, Newton, USA).

#### Adherence assay

Firstly, samples were stabilized in Mueller-Hinton Broth (MHB) for 30 min. Adjusted bacterial suspension was then added to the wells (10^7^ CFU/mL), and incubated at 37 °C for 2 h. Afterwards, samples were rinsed with sterile phosphate-buffered saline (PBS) solution and fixed with 4% (w/v) paraformaldehyde (PFA; Sigma–Aldrich) for 20 min. After rinsing with sterile PBS, samples were stained with VECTASHIELD® Mounting Medium with 40,6-diamidino-2-phenylindole (DAPI; Vector). Images were obtained with an inverted fluorescence microscope (Axiovert 200 M, Zeiss, Germany) (eight fields on each of triplicate replicates were obtained with a 1000x magnification, a net area ~0.1181 mm^2^/sample). ImageJ software manual counting mode was used to obtain bacterial counts. Also, supernatants were diluted, plated in triplicate on TSA and incubated at 37 °C for 18 h for colony forming units (CFU) counting. Results presented are representative of one of the three independent experiments with 3 replicates each performed.

#### Adherence assay in the presence of plasma proteins

Firstly, samples were stabilized in PBS or PBS with 1%(v/v) human plasma (provided by Centro Hospitalar de S. João, Porto) for 30 min. Adjusted bacterial suspension was then added to the wells (10^7^ CFU/mL), and incubated at 37 °C for 2 h. Afterwards, samples were rinsed with PBS solution and fixed with 4% (w/v) paraformaldehyde (PFA; Sigma–Aldrich) for 20 min. After rinsing with sterile PBS and samples were stained with VECTASHIELD® Mounting Medium with DAPI (Vector). Images were acquired as explained above. ImageJ software manual counting mode was used to obtain bacterial counts. Three replicates were used for each sample.

#### Proliferation assay

For proliferation assays, samples that were incubated during 2 h with bacterial suspension (as described in 5.4.3.) were rinsed with sterile PBS, and immersed in fresh MHB in a new 24-well plate for 4 h at 37 °C and 100 rpm. At the end of this period, surfaces were rinsed with sterile PBS, fixed and visualized as described above (5.4.3). Supernatants were also plated for CFU counting. Results presented are representative of one of the three independent experiments with 3 replicates each performed.

#### Bacterial Viability of adhered bacteria

To assess the viability of adherent bacteria in the different surfaces, a bacteria adherence assay was performed as described in section 5.4.3. After the 2 h incubation period samples were rinsed in NaCl 0.9% and stained with a combination dye of the LIVE/DEAD^®^ Bacterial Viability Kit (Baclight^TM^) for 15 min in the dark. Briefly, the kit contains two fluorescent dyes, Syto9 which stains all bacteria in green, and propidium iodide (PI) which can only crossover damaged cells membranes and gives red stained cells. As PI quenches the fluorescent emission of Syto9, it is assumed that green cells are alive whereas red cells are dead. Images were obtained as previously explained in section 5.4.3. Five replicates of each sample were analyzed by acquiring six fields of each replicate with a 630x magnification.

#### QCM-D Testing: Adsorption of proteins from human plasma

Gold-coated QCM-D sensors (fundamental frequency of 5 MHz) were purchased from Biolin Scientific. The sensors cleaning procedure consisted in a 10 min oxidation in a ultraviolet (UV) oven, followed by immersion in a “piranha” solution (3 parts hydrogen peroxide (H_2_O_2_) solution (Merck, 30% vol.) to 7 parts of Sulfuric acid (H_2_SO_4_) (Merck)). Afterwards, sensors were rinsed and sonicated for 3 min in type 2 water and dried with a gentle argon stream. After cleaning, the sensors were spincoated as previously explained in section 5.1.

QCM-D system (Q-Sense E4 instrument, Biolin Scientific) was used to monitor in real time the frequency (Δf) and dissipation (ΔD) shifts on the Au, Ch_Buffer and Ch_NAC4-QCM-D sensors related to protein adsorption. Briefly, samples were pre-incubated with PBS in static conditions for 30 min to establish a baseline. Afterwards, 500 μL/sensor of a 1% (v/v) of human plasma (provided by Centro Hospitalar de S. João, Porto) in PBS was injected in the system at a constant flow rate of 0.1 μL/min. After injection, incubation proceeded in static conditions for 2 h. Rinsing was performed with PBS, at a constant flow rate of 0.1 μL/min during 2 h. The entire experiment was conducted at 37 °C.

Data were treated using the Voigt model, since it takes in account the viscoelastic contributions of the hydrated layer. Data from the 3^rd^ to the 11^th^ harmonics were collected and used in the analysis. The density and viscosity of the human plasma solution was established at 1.35 g/cm^3^ and 0.0014 kg/ms^−1^, respectively^55,56^.

Results are presented in mass per area (ng/cm^2^). Results are average of 3 independent assays with 2 replicates per sample.

#### Scanning Electron Microscopy (SEM) visualization

Adherent bacteria were fixed with a freshly prepared solution of 1.5% glutaraldehyde (Merck) in 0.14 M sodium cacodylate (Merck) buffer for 30 min at room temperature. After fixation, samples were rinsed twice with PBS. Adherent bacteria were dehydrated in a graded ethanol series (50, 60, 70, 80, 90, and 99%, for 10 min each). Finally, 0.01 mL hexamethyldisilazane (Sigma) was added to each sample and left to dry overnight. Micrographs of the adhesion and proliferation assays were taken using a high resolution Scanning Electron Microscopy with X-ray Microanalysis, JEOL JSM 6301 F/Oxford INCA Energy 350 (from CEMUP – Centro de Materiais da Universidade do Porto). Electron beam intensity of 5 kV (accelerating voltage) and magnification of 1000x were applied. To increase the surface conductivity, samples were sputtered with Au/Pd for 60 s and 15 mA current using the SPI module sputter coater equipment.

#### Biofilm assay

*S. aureus* overnight culture in TSB (100 μL) was added to 5 mL of TSB-0.25% glucose and incubated for 4 h at 37 °C, 150 rpm. Then, 100 µL of this suspension were added to each well containing test surfaces already immersed in 900 µL of TSB-0.25% glucose. After a 2 h incubation period at 37 °C, surfaces were gently rinsed three times with sterile PBS and re-incubated with 1000 µL of TSB-0.25% glucose for 24 h. Finally, surfaces were rinsed with sterile PBS and left to dry. At this point, surfaces were stained with crystal violet 1% for 5 min and then washed with type 2 water. Crystal violet was solubilized in dimethyl sulfoxide (DMSO, Merck) and absorbance was read at 570 nm. Results are the average of three independent assays on each of three independent samples.

### Cellular Biocompatibility

#### Cell culture

MC3T3-E1 calvaria pre-osteoblast cells (purchased from the European Collection of Cell Cultures - ECACC) were cultured in complete α-modified minimal essential medium (α-MEM, Gibco) supplemented with 10% (v/v) heat-inactivated fetal bovine serum (FBS; Gibco) and 1% (v/v) Penicillin/Streptomycin (P/S; Gibco). The cells were maintained at 37 °C in humidified atmosphere of 5% CO_2_, and media were refreshed every 2 to 3 days. At pre-confluence, MC3T3-E1 cells were harvested using trypsin solution (0.25% w/v trypsin, 0.1% w/v glucose and 0.05% w/v ethyldiaminetetracetic acid in PBS) and were plated on top of chitosan and chitosan-modified films (n = 5) at a density of 2.0 × 10^4^ cells, under osteogenic conditions (complete α-MEM supplemented with 50 µg/ml ascorbic acid and 10 mM β-glycerophosphate). Multiwells culture plates were previously coated with polyhydroxyethylmethacrylate (pHEMA) at a final density of 0.8 mg/cm^2^ to avoid adhesion of the cells to the bottom and walls of the well. Tissue culture coverslips were used as positive controls. All experiments were performed using cells until passage 13.

#### Cytotoxicity assays

Cell metabolic activity and morphology were evaluated at 3, 7 and 14 days of culture. Cell metabolic activity was analysed by the Resazurin assay. Briefly, cells were incubated with 10% (v/v) Resazurin solution (0.1 mg/mL; Sigma–Aldrich) for 4 h at 37 °C. After incubation, three 150 μL/well samples were transferred to a 96-well black plate and fluorescence was measured (530 nm Ex/590 nm Em) in a microplate reader (BioTek Synergy Mx, Molecular Devices). Cell morphology was assessed by staining the filamentous actin cytoskeleton of the cells, through immunocytochemistry. For that, medium was removed from the wells, and cells were washed twice with PBS at room temperature (RT) and fixed in 4% (w/v) PFA (Sigma–Aldrich) for 10 min. Cells were then washed with PBS and permeabilized for 5 min with ice-cold 0.1% (v/v) Triton X-100 in PBS. After one PBS rinse, samples were incubated with 1% (w/v) bovine serum albumin (BSA) (Gibco, Alfagene) for 30 min at 37 °C. Afterwards, samples were incubated with Alexa Fluor® 594 phalloidin antibody (1:100; Molecular Probes, Invitrogen) in PBS for 20 min in the dark at RT. Samples were washed twice with PBS to remove unbound antibody, and mounted with VECTASHIELD® with DAPI. Samples were protected from light and kept at −20 °C until further analysis by inverted fluorescence microscopy (Zeiss AxioVision Microscope).

### Statistical analysis

For statistical analysis, one-way analyses of variance followed by Tukey’s post hoc testing were used. The non-parametric Kruskal–Wallis test was applied when Gaussian distribution was not confirmed. Analyses were performed using the GraphPad Prism program. Data are expressed as the mean ± standard deviation (SD), and p values of <0.05 were considered significant. Data available on request from the authors.

## Electronic supplementary material

Supplementary data
