# Supplementary material for: N-acetylcysteine-functionalized coating avoids bacterial adhesion and biofilm formation
Source: Sci Rep. 2017 Dec 12;7:17374. doi: 10.1038/s41598-017-17310-4 (PMC5727138; doi:10.1038/s41598-017-17310-4)
Supplement: Supplementary file 1 — Supplementary data [file 41598_2017_17310_MOESM1_ESM.pdf]

Supplementary Data

# N-acetylcysteine-functionalized coating avoids bacterial adhesion and biofilm formation

Fabíola Costa [a,b], Daniela M. Sousa [a,b], Paula Parreira [a,b], Meriem Lamghari [a,b], Paula Gomes [c], M. Cristina L. Martins [a,b,d*]

[a] i3S, Instituto de Investigação e Inovação em Saúde, Universidade do Porto, Portugal, Rua
[b] INEB - Instituto de Engenharia Biomédica, Universidade do Porto, Porto, Portugal
[c] UCIBIO-REQUIMTE, Departamento de Química e Bioquímica, Faculdade de Ciências, Universidade do Porto, Porto, Portugal
[d] Universidade do Porto, Instituto de Ciências Biomédicas Abel Salazar, Porto, Portugal

* Corresponding author: M. Cristina L. Martins
INEB - Instituto de Engenharia Biomédica; Rua Alfredo Allen, 308, 4200-135 Porto, Portugal
Tel: +351 22 6074982
E-mail address: cmartins@ineb.up.pt

(SSD) Table 1. Theoretical surface atomic composition (%) assuming full chitosan functionalization.

| Theoretical at % | Ch | Ch_EDC | Ch_NAC |
|---|---|---|---|
| *C1s* | 55 | 64.4 | 53 |
| *N1s* | 9 | 16.4 | 10 |
| *O1s* | 36 | 19.2 | 32 |
| *S2p* | 0 | 0 | 5 |

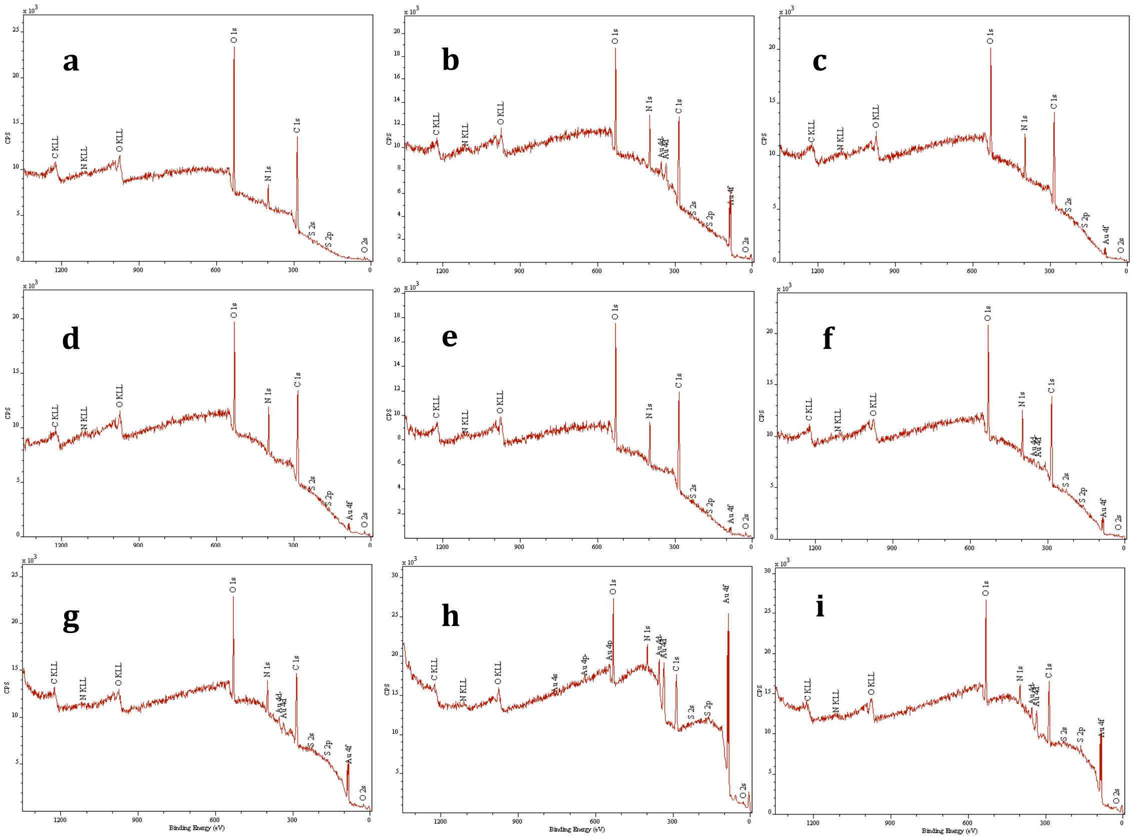

SSD Fig.1. XPS survey spectra for chitosan and chitosan-modified films: (a) Ch_Buffer, (b) Ch_EDC, (c) Ch_NAC0.4, (d) Ch_NAC2, (e) Ch_NAC4, (f) Ch_NAC8, (g) Ch_NAC12, (h) Ch_NAC16, and (i) Ch_NAC20.

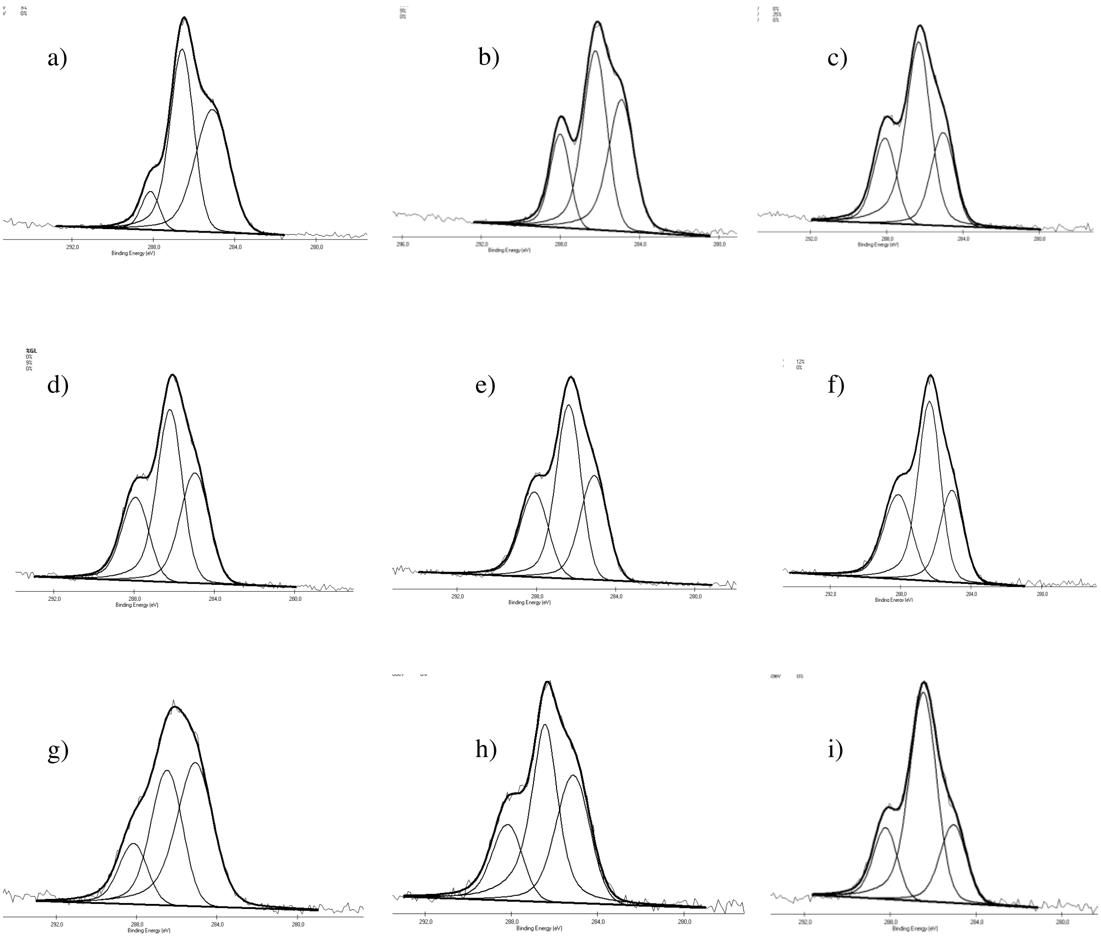


SSD Fig.2. C1s high-resolution XPS spectra for chitosan and chitosan-modified films: (a) Ch_Buffer, (b) Ch_EDC, (c) Ch_NAC0.4, (d) Ch_NAC2, (e) Ch_NAC4, (f) Ch_NAC8, (g) Ch_NAC12, (h) Ch_NAC16, and (i) Ch_NAC20. Peaks at ~285 eV correspond to C-C ligation, peaks at ~286.4 eV correspond to C-OH/C-O-C ligations, peaks at 287.9 eV are assigned to C=N and peaks at 288.1 eV correspond to O-C-O/N-C=O.

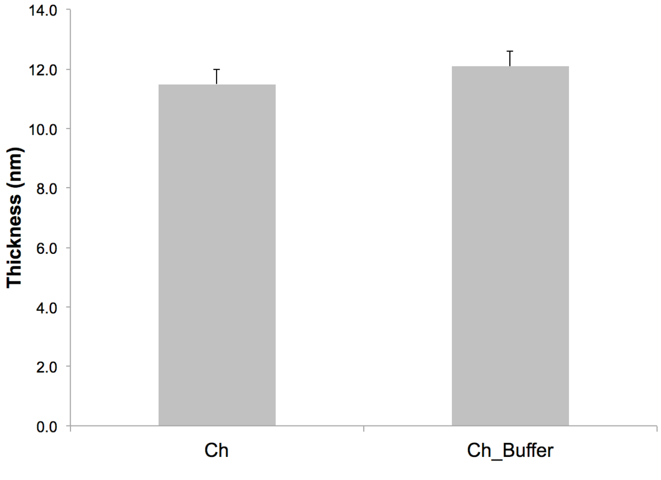


SSD Fig.3. Chitosan films thickness measurements before and after incubation with NAC-immobilization buffer (MES Buffer). Three replicates of each sample were used.

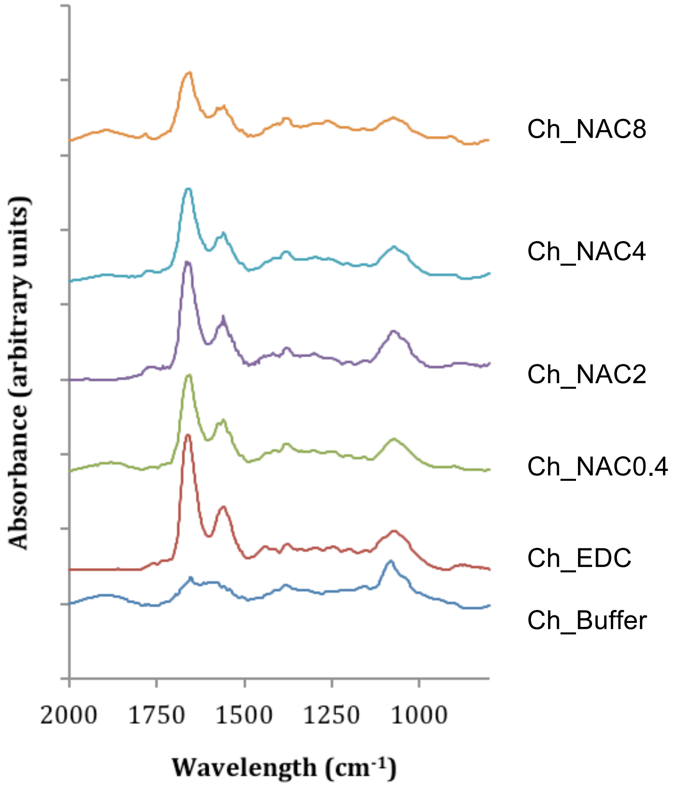


SSD Fig.4. IRRAS spectra of chitosan and chitosan-modified films.

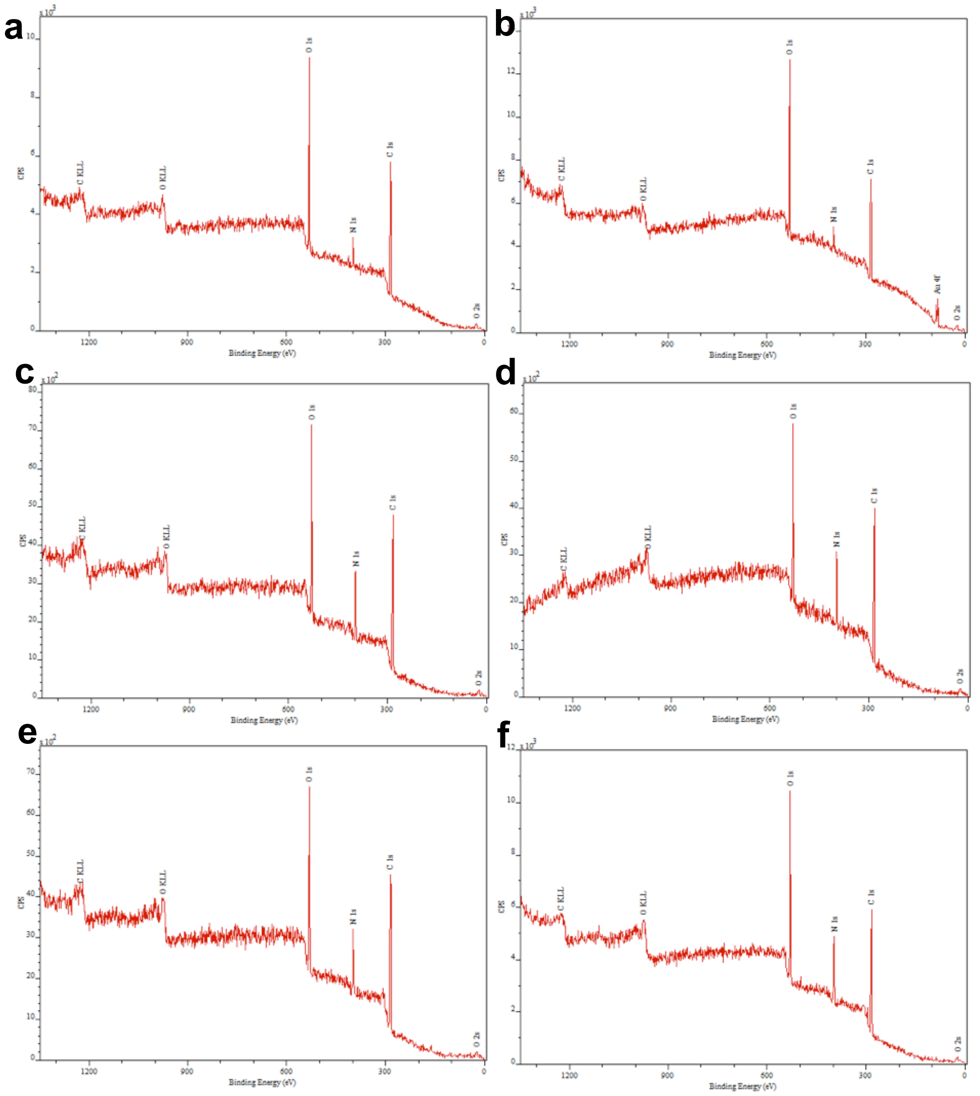


SSD Fig.5. XPS survey spectra for chitosan and chitosan-modified films before and after incubation in PBS at 37ºC for 7 days: (a) Ch_Buffer, day 0; (b) Ch_Buffer, day 7; (c) Ch_NAC4, day 0; (d) Ch_NAC4, day 7; (e) Ch_NAC8, day 0; (f) Ch_NAC8, day 7.

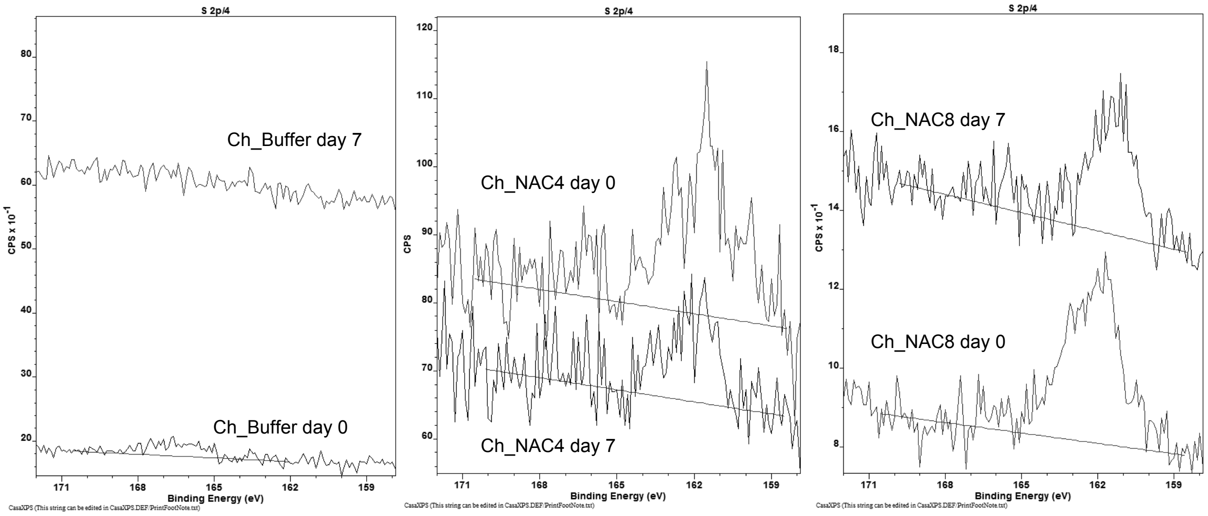


SSD Fig.6. XPS S2p High-resolution spectra for chitosan and chitosan-modified films before and after incubation in PBS at 37ºC for 7 days.
